# Development of a Radiofluorinated Adenosine A_2B_ Receptor Antagonist as Potential Ligand for PET Imaging

**DOI:** 10.3390/ijms21093197

**Published:** 2020-04-30

**Authors:** Marcel Lindemann, Rareş-Petru Moldovan, Sonja Hinz, Winnie Deuther-Conrad, Daniel Gündel, Sladjana Dukic-Stefanovic, Magali Toussaint, Rodrigo Teodoro, Cathleen Juhl, Jörg Steinbach, Peter Brust, Christa E. Müller, Barbara Wenzel

**Affiliations:** 1 Department of Neuroradiopharmaceuticals, Institute of Radiopharmaceutical Cancer Research, Helmholtz-Zentrum Dresden-Rossendorf, 04318 Leipzig, Germany; marcel.lindemann@uit.no (M.L.); r.moldovan@hzdr.de (R.-P.M.); w.deuther-conrad@hzdr.de (W.D.-C.); d.guendel@hzdr.de (D.G.); s.dukic-stefanovic@hzdr.de (S.D.-S.); m.toussaint@hzdr.de (M.T.); r.teodoro@hzdr.de (R.T.); j.steinbach@hzdr.de (J.S.); p.brust@hzdr.de (P.B.); 2 Pharma Center Bonn, Pharmaceutical & Medicinal Chemistry, Pharmaceutical Institute, University of Bonn, 53121 Bonn, Germany; Sonja.Hinz@uni-wh.de (S.H.); christa.mueller@uni-bonn.de (C.E.M.); 3 ROTOP Pharmaka GmbH, 01328 Dresden, Germany; service@rotop-pharmaka.de

**Keywords:** A_2B_ adenosine receptor, adenosine, PET, fluorine-18, metabolism, radiofluorination

## Abstract

The adenosine A_2B_ receptor has been proposed as a novel therapeutic target in cancer, as its expression is drastically elevated in several tumors and cancer cells. Noninvasive molecular imaging via positron emission tomography (PET) would allow the in vivo quantification of this receptor in pathological processes and most likely enable the identification and clinical monitoring of respective cancer therapies. On the basis of a bicyclic pyridopyrimidine-2,4-dione core structure, the new adenosine A_2B_ receptor ligand **9** was synthesized, containing a 2-fluoropyridine moiety suitable for labeling with the short-lived PET radionuclide fluorine-18. Compound **9** showed a high binding affinity for the human A_2B_ receptor (*K*_i_(A_2B_) = 2.51 nM), along with high selectivities versus the A_1_, A_2A_, and A_3_ receptor subtypes. Therefore, it was radiofluorinated via nucleophilic aromatic substitution of the corresponding nitro precursor using [^18^F]F^-^/K_2.2.2._/K_2_CO_3_ in DMSO at 120 °C. Metabolic studies of **[^18^F]9** in mice revealed about 60% of radiotracer intact in plasma at 30 minutes p.i. A preliminary PET study in healthy mice showed an overall biodistribution of **[^18^F]9**, corresponding to the known ubiquitous but low expression of the A_2B_ receptor. Consequently, **[^18^F]9** represents a novel PET radiotracer with high affinity and selectivity toward the adenosine A_2B_ receptor and a suitable in vivo profile. Subsequent studies are envisaged to investigate the applicability of **[^18^F]9** to detect alterations in the receptor density in certain cancer-related disease models.

## 1. Introduction

Adenosine receptors belong to the family of G-protein-coupled receptors and are activated by the purine nucleoside adenosine, a precursor and degradation product of adenine nucleotides such as adenosine 5′-triphosphate (ATP). The effects of adenosine are mediated via the four receptor subtypes A_1_, A_2A_, A_2B_, and A_3_. Each of these exhibits distinct pharmacological properties, cell and tissue distribution, and intracellular signaling [1,2,3]. The adenosine A_2B_ receptor can be seen as unique as its activation requires high levels of adenosine with concentrations in the micromolar range [3,4,5], accompanied by an ubiquitous expression at mostly moderate to low levels [3,6]. Therefore, in comparison with the other adenosine receptors, the function and physiological role of this low-affinity receptor has been sparsely characterized to date and is barely understood. However, in recent years, the involvement of the A_2B_ receptor in pathophysiological processes was discovered to be driven by the fact that extracellular concentrations of adenosine increase under certain pathological conditions such as hypoxia or inflammation [6,7,8]. In particular, the relevance of A_2B_ receptor in promoting cancer progression has been revealed via mechanisms such as tumor growth, angiogenesis, metastasis, and immunomodulation, and it has been shown that its expression can be drastically increased in tumors and cancer cells, as recently reviewed [6,9,10,11]. Moreover, current pharmaceutical studies indicate that the A_2B_ receptor might be particularly appropriate for cancer therapies in comparison to other adenosine receptor subtypes that show to some extend both pro- and antitumor effects [7,9,12]. Thus, the adenosine A_2B_ receptor is a highly relevant therapeutic target, although extensive and interdisciplinary research is still needed to gain a deeper understanding.

**Figure 1:**
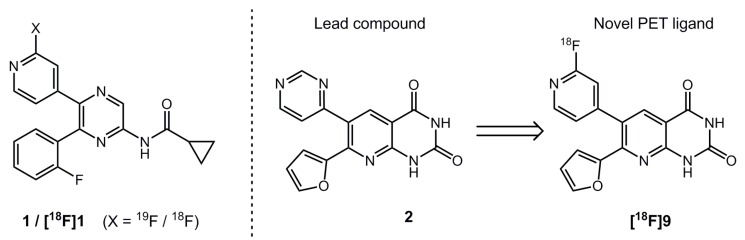
Molecular structures of the previously radiofluorinated PET ligand **[^18^F]1** [14], the selected new bicyclic lead compound **2** [16], and the newly developed A_2B_ receptor PET ligand **[^18^F]9**.

Noninvasive molecular in vivo imaging using positron emission tomography (PET) could be one tool used to approach this goal. For example, it would allow quantification of the A_2B_ receptor in pathological processes and probably enable clinical monitoring of A_2B_-receptor-related cancer therapies. Therefore, the development of a suitable radioligand to target the adenosine A_2B_ receptor with high affinity and selectivity, accompanied by a suitable pharmacokinetic and metabolic profile in vivo is desired. In 2016, Petroni et al. [13] developed the first PET radiotracer for this receptor subtype, which was labeled with the short-lived radionuclide carbon-11 (*t*_1/2_ = 20.4 min). The authors investigated the compound in rats and concluded that it might represent a good candidate, but further characterization would be needed. We recently reported on the first PET radiotracer (**[^18^F]1**, Figure 1) labeled with the radionuclide fluorine-18 (*t*_1/2_ = 109.8 min) [14]. The radiotracer was designed using structural modification on the basis of a monocyclic pyrazine compound class published by Eastwood, Vidal, et al. [15], and resulted in a small series of fluorinated derivatives. The most appropriate candidate, **1**, showed a high binding affinity for the A_2B_ receptor (*K*_i_ = 4.24 nM). Despite its moderate selectivity (see data in Table 1), we synthesized the corresponding radiotracer **[^18^F]1** for investigation of the pharmacokinetic profile of this monocyclic compound class in vivo. Rapid metabolic degradation of **[^18^F]1** was observed in mice, with the formation of a radiometabolite generated by the cleavage of the amide bond which, besides the moderate selectivity, might cause difficulties for specific imaging.

**Scheme 1:**
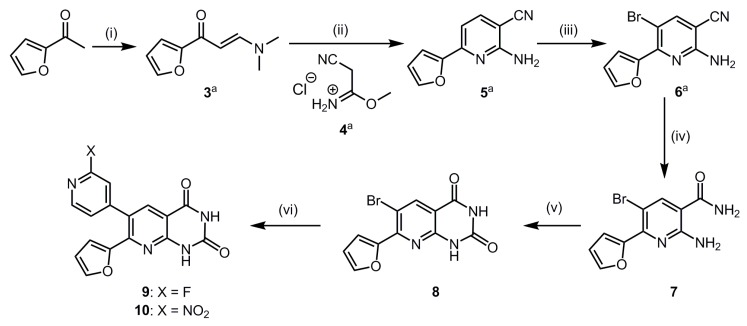
Synthesis of the new A_2B_ receptor ligand **9** and its precursor for radiofluorination **10**. Reaction conditions: (i) *N,N*-dimethylformamide dimethyl acetal, reflux, 5.5 h, 90%, (ii) 4, NH_4_OAc, MeOH, reflux, 12 h, 70%, (iii) *N*-bromosuccinimide (NBS), *N,N*-dimethylformamide (DMF), 12 hours, rt, 77%, (iv) 6 M NaOH, H_2_O_2_, 50 °C, 5 h, 45%; (v) a) NaH, DMF, 30 min, rt, b) *N,N*-carbonyldiimidazole, 90 °C, 2 h, 50%; (vi) **9**: 2-fluoro pyridine-4-boronic acid, 2 M Cs_2_CO_3_, [Pd(dppf)Cl_2_], 1,4-dioxane, 90 °C, 2 h, 59%; **10**: (a) 4-bromo-2-nitropyridine, 4,4,4’,4’5,5,5’,5’-octamethyl-2,2’-bis(1,3,2-dioxaborolane), KOAc and [Pd(dppf)Cl_2_], 90 °C, 1 h (b) 2 M Cs_2_CO_3_, [Pd(dppf)Cl_2_], 90 °C, 1 h, 9%. ^a^ The synthesis of compounds **3**–**6** has been previously reported [18,19,20] and was performed accordingly with slight modifications, as described in the Supplementary material.

Therefore, the aim of the present study was to develop a new radiofluorinated PET tracer possessing (i) a higher selectivity for the adenosine A_2B_ receptor versus the other subtypes, and (ii) a suitable metabolic profile in vivo. For this purpose, we selected the bicyclic pyridopyrimidine-2,4-dione core as the lead structure (**2** in Figure 1), which was previously described to fulfill both criteria [16]. The authors reported for **2** a *K*_i_ value of 1 nM at the A_2B_ receptor (see Table 1) and a high metabolic stability, determined via in vitro studies with rat hepatic microsomes [16]. In order to introduce the required radiofluoro atom, the pyrimidine ring of **2** was substituted by a 2-fluoropyridine ring representing a very common heteroaromatic motive for ^18^F-radiolabeling reactions (**[^18^F]9**, Figure 1). For a straightforward radiosynthesis using nucleophilic aromatic substitution with [^18^F]fluoride, a precursor compound was developed containing a nitro group as a suitable leaving group (**10**, Scheme 1).

This communication describes (i) the organic and radiosynthesis of the new A_2B_ receptor PET ligand **[^18^F]9**, (ii) the in vitro binding data of the non-radioactive ligand **9** toward all four adenosine receptor subtypes, and (iii) preliminary results for **[^18^F]9** regarding in vivo metabolism and biodistribution.

## 2. Results and Discussion

### 2.1. Organic Synthesis and In Vitro Binding Studies

In order to investigate the influence of the substitution of the pyrimidine ring in lead compound **2** by the 2-fluoropyridine ring in target compound **9** on the affinity and selectivity toward the A_2B_ receptor, **2** was synthesized as a reference compound. This synthesis was performed according to the procedures described by Eastwood et al. [16,17]. For the synthesis of the new A_2B_ receptor ligand **9**, another strategy was chosen, which is shown in Scheme 1. Briefly, the three-fold substituted pyridine core **5** was built up via a two-step synthesis sequence starting from 2-acetyl furane, which was treated with *N,N*-dimethylformamide dimethyl acetal to obtain compound **3** [18]. Further reaction of **3** with the hydrochloride salt **4** and ammonium acetate gave **5**, which was selectively brominated to obtain compound **6**. Derivatives **3**–**6** have been previously described and the reported synthesis procedures [18,19,20] were adopted with slight modifications (see Supplementary material). In the next step, the cyano group was hydrolyzed using hydrogen peroxide under basic conditions to obtain the amide **7**, which was then cyclized to **8** by deprotonation with sodium hydride and subsequent reaction with *N,N*-carbonyldiimidazole. The obtained pyridopyrimidine-2,4-dione **8** was used for a Suzuki coupling reaction [21,22] with 2-fluoro pyridine-4-boronic acid to achieve the desired compound **9**. The bicyclic intermediate **8** was also used for the synthesis of the precursor compound **10**. The required 2-nitropyridine-4-boronic acid pinacol ester was synthesized via a Miyaura borylation [23] of the corresponding bromo-substituted nitropyridine and subsequently coupled in situ with **8** in a Suzuki reaction, affording the nitro precursor **10**.

The binding affinities of **2** and **9** at the four human adenosine receptor subtypes A_2B_, A_2A_, A_1_, and A_3_, presented in Table 1, were determined in competitive binding assays using appropriate radioligands and membrane preparations of Chinese hamster ovary (CHO) cells or human embryonic kidney (HEK) cells, respectively, recombinantly expressing the corresponding human receptor subtype. 

The in-house-determined binding data of compound **2** corresponded to the values reported by Eastwood et al. [16] and thus confirmed its high affinity and selectivity for the A_2B_ receptor. The substitution of the pyrimidine ring in **2** by a 2-fluoropyridine ring in the new derivative **9** did not affect the binding affinity for the A_2B_ receptor, as indicated by the *K*_i_ value of 2.51 nM obtained for both compounds. Accordingly, compound **9** possesses a two-fold higher binding affinity for the A_2B_ receptor in comparison to our recently developed ligand **1**. Moreover, the selectivity of **9** regarding the two adenosine receptor subtypes A_1_ and A_2A_ was considerably higher than that of **1** (*K*_i_ ratios of 43 and 59 versus 13 and 4.5, resp.; see Table 1). Due to a slight increase in the binding affinity of **9** for the A_3_ receptor, the selectivity regarding this off-target was slightly decreased but still in a suitable range for the purpose of specific imaging (*K*_i_ ratio of 114).

### 2.2. Automated Radiosynthesis and Characterization of **[^18^F]9**

**Scheme 2:**
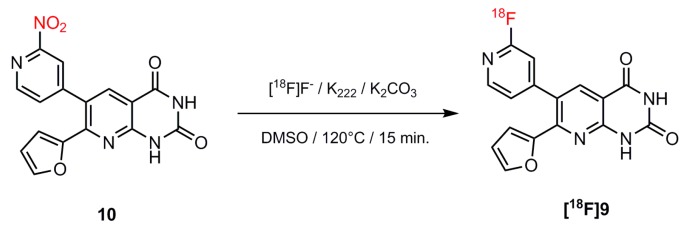
^18^F-labeling of precursor **10** to obtain **[^18^F]9.**

The radiolabeling of **[^18^F]9** was performed via a nucleophilic aromatic substitution reaction of the nitro group of precursor **10** and [^18^F]fluoride (Scheme 2), which was azeotropically dried using the kryptofix/potassium carbonate system and acetonitrile (ACN). Based on our former experiments with compound **[^18^F]1** [14], we selected dimethyl sulfoxide (DMSO) as the most promising solvent. All radiolabeling experiments were performed with a very low amount of precursor (about 0.5 mg).

The complete radiosynthesis of **[^18^F]9** was accomplished in a remotely controlled synthesis module (TRACERlab FX2 N from GE Healthcare). The synthesizer setup is described in detail in the Materials and Methods. Briefly, [^18^F]fluoride was trapped on an anion exchange cartridge, eluted with aqueous potassium carbonate, and then azeotropically dried to obtain the [^18^F]F^−^/K_2.2.2._/K_2_CO_3_ complex. Radiofluorination of the precursor **10** was performed in DMSO for 15 min at 120 °C. In order to isolate **[^18^F]9**, the crude reaction mixture was diluted with aqueous methanol and applied to a semi-preparative HPLC system (chromatogram in Figure S1 in Supplementary Material). The radiotracer fraction was collected at retention times in the range of 22–25 min and purified by solid-phase extraction using a C18 cartridge. Afterwards, **[^18^F]9** was eluted from the cartridge and transferred out of the hot cell for concentration and formulation in sterile isotonic saline containing 10% ethanol. The entire process lasted about 80 min. With this procedure, **[^18^F]9** could be reproducibly generated with a high radiochemical purity of ≥99%, reasonable radiochemical yields of 48% ± 4% (*n* = 4) and molar activities of 10–32 GBq/µmol (*n* = 4) at starting activities of 2–7 GBq. The identity of **[^18^F]9** was confirmed by analytical radio-HPLC (Figure S2 in Supplementary Material) with co-injection of the corresponding non-radioactive reference compound **9**.

For estimation of the lipophilicity of **[^18^F]9**, the shake-flask method with *n*-octanol and phosphate-buffered saline as a partition system was used, resulting in a logD value of 1.4 ± 0.1 (*n* = 4).

### 2.3. In Vivo Studies of **[^18^F]9** in Mice

The metabolism of **[^18^F]9** in vivo was investigated in CD-1 mice by taking blood and urine samples at 30 min post i.v. injection of the radiotracer (*n* = 2). Analysis of the respective samples was performed by micellar (MLC) and reversed-phase chromatography (RP-HPLC) as two complementary HPLC methods. MLC is also a reversed-phase HPLC mode, but it uses a mobile phase consisting of an aqueous solution with a surfactant above its critical micellar concentration. The formed micelles are able to dissolve the proteins and other components of a biological sample and therefore enable a direct injection of the plasma probe into the HPLC system without eliminating the tissue matrix [24]. In contrast, for RP-HPLC analysis, aliquots of the plasma samples were treated with a mixture of ACN/water to precipitate the proteins, resulting in recoveries of activity of about 93%.

**Figure 2:**
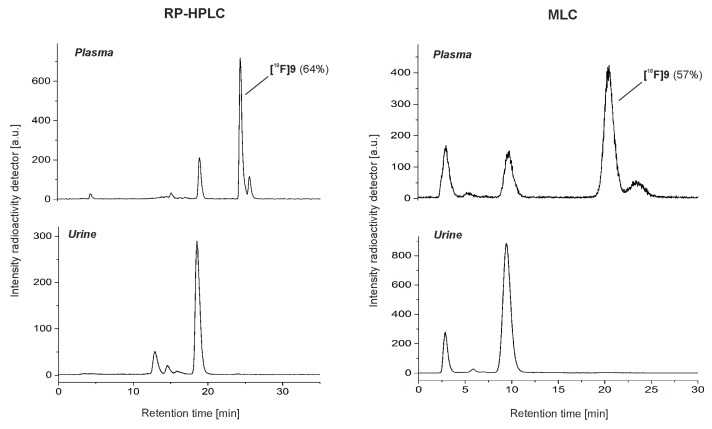
Exemplary RP-HPLC (left) and MLC (right) radio-chromatograms of mouse plasma and urine samples at 30 min p.i. of **[^18^F]9** (conditions RP-HPLC: Reprosil-Pur C18-AQ, 250 × 4.6 mm, gradient with eluent mixture of ACN/20 mM aq. NH_4_OAc, 1.0 mL/min; conditions MLC: Reprosil-Pur C18-AQ, 250 × 4.6 mm, gradient with an eluent mixture of EtOH/100 mM aq. SDS/25 mM aq. (NH_4_)_2_HPO_4_, 1.0 mL/min).

In general, the results obtained with both methods were comparable. Figure 2 shows exemplary RP-HPLC and MLC chromatograms of a plasma and urine sample at 30 min p.i. Accordingly, intact radiotracer accounted for ~60% of the total activity in plasma, which was a considerably higher fraction of intact radiotracer in comparison to our former radiotracer **[^18^F]1** (35% at 30 min p.i.) [14]. The main radiometabolite was slightly more polar than **[^18^F]9** and was also found in the urine, indicating, along with a very high activity concentration (standardized uptake value (SUV) = 75), a renal excretion route for **[^18^F]9**. Besides this main radiometabolite, a small fraction of more polar radioactive metabolites was observed in urine.

When comparing the chromatograms of the two HPLC methods, slight differences in the retention profile became evident, a phenomenon that is caused by the differences in the separation mechanisms of the two chromatographic systems. Moreover, the RP-HPLC chromatograms showed a lower fraction of a very polar radiometabolite (within the first 5 min retention time) compared to the MLC ones. Considering the plasma sample, this influenced the peak quantification and therefore resulted in a supposed slightly higher percentage of intact radiotracer using the RP-HPLC method. However, it is likely that this polar component could not be quantitatively extracted during the plasma work-up procedure used to generate the injection sample for RP-HPLC, as reflected by the non-quantitative recovery of 93%. Considering this small loss resulted in a calculated value of 60% of intact tracer for RP-HPLC, which was comparable to the MLC result. In a separate in vitro experiment, the quantitative recovery of the radiotracer could be shown using the same work-up procedure.

**Figure 3:**
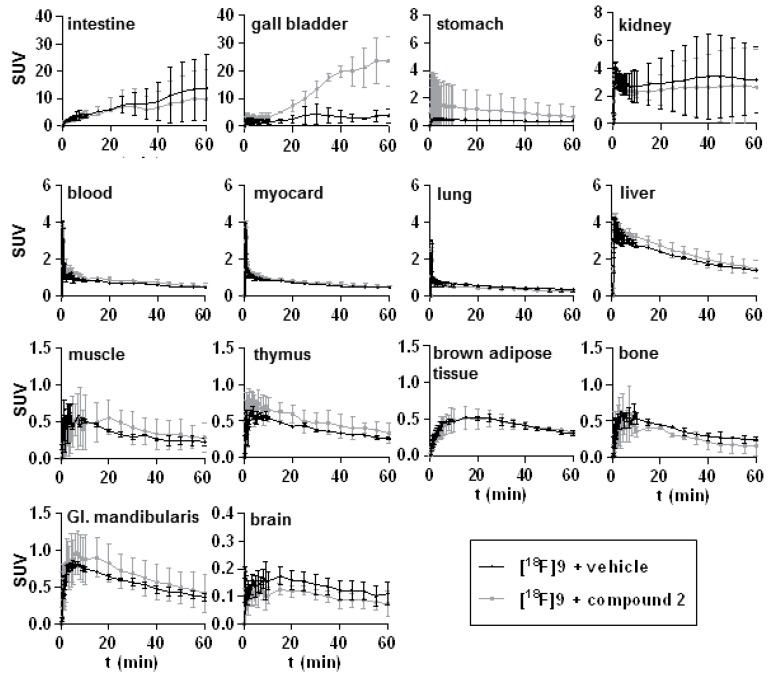
Biodistribution of **[^18^F]9** in female CD-1 mice derived by small-animal PET over 60 min; time–activity curves (TACs) for tissue uptake are expressed as standard uptake values (SUVs), *n* = 2, mean ± SD.

We then performed a pilot study to estimate very basic characteristics of the pharmacokinetics of the new radiotracer via dynamic small-animal PET imaging with **[^18^F]9** in female CD-1 mice. The time–activity curves (TACs) obtained in different tissues under both baseline (administration of vehicle, *n* = 2) and blocking (administration of compound **2**, *n* = 2) conditions are presented in Figure 3; the whole-body maximum intensity projections are shown in Figure S3 in the Supplementary Material. The overall biodistribution of **[^18^F]9** under baseline conditions corresponded to the known quite ubiquitous but physiologically not very dense expression of A_2B_ receptors [25]. Although higher levels of the A_2B_ receptor protein have been reported in the literature for certain cell types, such as alveolar epithelial cells and endothelial cells [3,25], the resemblance of the TACs in peripheral organs such as lungs under baseline and blocking conditions indicated no target-specific retention of **[^18^F]9** (TAC peak SUV _lung_: 3.09 and 2.88 vs. 2.30 and 1.57, resp.; SUV_60 min p.i., lung_: 0.44 and 0.25 vs. 0.32 and 0.20, resp.). This was slightly different to the results of Petroni et al. obtained for the carbon-11-labeled compound **[^11^C]-4** in rats [13], because those authors described a significant decrease of uptake of **[^11^C]-4** in the lung induced by the pretreatment of the A_2B_ receptor agonist BAY60-6583. This discrepancy between our and Petroni’s results might indicate differences in the biological effects exerted by agonistic (BAY60-6583) or antagonistic (compound **2**) A_2B_ receptor ligands, as well as model-related differences such as the expression of A_2B_ receptors. The latter option could also explain the about 10-fold lower uptake of **[^18^F]9** in the brown adipose tissue (BAT) of female CD-1 mice in comparison to the uptake of **[^11^C]-4** in the BAT of male Wistar rats [13].

The time–activity distribution in the kidney, along with the concentration of activity determined in the urine during the metabolism study, indicated a renal excretion route of the radiotracer. In addition, the remarkably high and constantly increasing uptake determined in the gall bladder under blocking conditions indicated accumulation of **[^18^F]9** in the bile, promoted by compound **2**. A low tracer accumulation was detected in the brain, with an initial TAC peak SUV of 0.19 (0.20 and 0.17) and subsequent slow washout to an SUV of 0.11 (0.14 and 0.08) after 60 min p.i. Co-administration of compound **2** resulted in a slight reduction of the brain uptake, with a TAC peak SUV of 0.12 (0.11 and 0.13).

Although this study was performed in healthy animals, thus in an animal model not characterized by disease-related high expressions of A_2B_ receptors, these initial results are promising regarding a low non-specific binding of **[^18^F]9** under physiological conditions. Accordingly, **[^18^F]9** seems to be applicable for imaging of A_2B_ receptors in future studies in disease models, e.g., of inflammation or cancer. However, further studies are required in order to answer open questions such as the unexpected increase of activity in the gall bladder after pretreatment with antagonist **2**.

## 3. Materials and Methods

### 3.1. Organic Chemistry

#### 3.1.1. General

Analysis of all compounds was performed via mass spectroscopy (MS), thin-layer chromatography (TLC), and NMR spectroscopy. The final compounds **2**, **9**, and **10** were also subjected to HPLC analysis.

High-resolution mass spectra were recorded on an ESI-qTOF Impact II (Bruker Daltonik GmbH, Leipzig, Germany) and on an ESI-TOF micrOTOF (Bruker Daltonik GmbH, Leipzig, Germany) using ElectroSpray Ionization (ESI). NMR spectra (^1^H, ^13^C, ^13^C-APT, ^19^F, H,H-COSY, HSQC, HMBC) were recorded on spectrometers from Varian and Bruker. Splitting patterns are designated herein as follows: s: singlet, d: doublet, bs: broad singlet, dd: doublet of doublet.

Analytical TLC was performed on silica gel-coated plates (ALUGRAM SIL G/UV_254,_ Machery-Nagel, Düren, Germany). The spots were identified using a UV lamp or by spraying a solution of 0.1% ninhydrin in ethanol/water 1/10 or dipping into a KMnO_4_ solution (3 g KMnO_4_, 20 g K_2_CO_3_, 0.25 ml glacial acid, 300 ml water).

For purification of final products, flash-column chromatography was used with silica gel ZEOsorb 60/40-63 µm from Apollo Scientific Ltd., Cheshire, UK and silica gel 40–63 µm from VWR International GmbH, Darmstadt, Germany.

The chemical purity of the final compounds was ≥ 96% and was controlled by HPLC using a 150 × 3 mm Reprosil-Pur Basic HD 3 µm column (Dr. Maisch HPLC GmbH, Ammerbuch-Entingen, Germany). These analytical chromatographic separations were performed on a Dionex Ultimate 3000 system, incorporating a LPG-3400SD pump, an autosampler WPS-3000 TSL, a column compartment TCC-3000SD, a diode array detector DAD3000 (monitoring from 254–720 nm), and a low-resolution mass spectrometer MSQ 3000 (Thermo Fisher Scientific, Darmstadt, Germany). A mixture of acetonitrile (ACN) and aqueous 20 mM NH_4_OAc was used as eluent in a linear gradient system (0–2.5 min at 25% ACN, 2.5–11.0 min up to 95% ACN, 11.0–13.0 min at 95% ACN, 13.0–13.5 min up to 25% ACN, 13.5–15.0 min at 25%) with a flow of 0.6 mL/min.

Chemical names of compounds were generated using ChemDraw Professional 17.0 (PerkinElmer Informatics, Waltham, MA, USA).

#### 3.1.2. 7-(Furan-2-yl)-6-(pyrimidin-4-yl)pyrido[2,3-*d*]pyrimidine-2,4-(1*H*,3*H*)-dione (**2**)

Compound **2** was synthesized according to the procedures reported by Eastwood et al. [16,17].

^1^H NMR (DMSO-*d_6_*, 300 MHz) *δ*: 6.60 (dd, *J* = 3.5, 1.7 Hz, 1 H), 6.86 (dd, *J* = 3.5, 0.8 Hz, 1 H), 7.48 (dd, *J* = 5.2, 1.4 Hz,, 1 H), 7.66 (dd, *J* = 1.7, 0.8 Hz, 1 H), 8.28 (s, 1 H), 8.81 (d, *J* = 5.2 Hz, 1H), 9.21 (d, *J* = 1.3 Hz, 1 H), 11.52 (s, 1H), 11.89 (s, 1H) ppm. ^13^C NMR (DMSO-*d_6_*, 75 MHz) *δ*: 108.9, 112.9, 114.8, 121.9, 126.2, 139.5, 146.0, 150.1, 150.9, 151.6, 152.7, 157.6, 158.9, 162.1, 164.2 ppm. HRMS (ESI+) *m/z* calcd. for C_15_H_10_O_3_N_5_^+^ [M+H]^+^ 308.07782, found 308.07765.

#### 3.1.3. Compounds **3**–**6**

These compounds were synthesized as described in the Supplementary Material.

#### 3.1.4. 2-Amino-5-bromo-6-(furan-2-yl)nicotinamide (**7**)

A suspension of compound **6** (0.74 g, 2.8 mmol) in 22 mL ethanol, 15 mL water, 9.3 mL 6M aqueous NaOH and 0.8 mL H_2_O_2_ (50% in H_2_O, 14.0 mmol) was stirred for 5 h at 50 °C. Afterwards, 200 mL water was added and the mixture was extracted with ethyl acetate (4 × 25 mL). The combined organic phases were dried over sodium sulfate, filtered, and the solvent was removed under reduced pressure. The obtained residue was purified by column chromatography (SiO_2_, ethyl acetate/*n*-hexane, 2/1, *v*/*v*) to obtain **7** with a yield of 45% (0.36 g, 1.26 mmol).

^1^H NMR (DMSO-*d_6_*, 400 MHz) *δ*: 6.69 (dd, *J* = 3.5, 1.8 Hz, 1 H), 7.35 (dd, *J* = 3.5, 0.8 Hz, 1 H), 7.46 (bs, 3 H), 7.90 (d, *J* = 0.9 Hz, 1 H), 8.09 (bs, 1 H); 8.28 (s, 1H) ppm. ^13^C NMR (DMSO-*d_6_*, 100 MHz) *δ*: 100.1, 109.5, 112.3, 114.6, 142.9, 145.0, 147.3, 151.0, 157.6, 168.6 ppm. HRMS (ESI+) *m/z* calcd. for C_10_H_9_O_2_N_3_^79^Br^+^ [M+H]^+^ 281.98727, C_10_H_8_O_2_N_3_^79^BrNa^+^ [M+Na]^+^ 303.96921, found 281.98678, 303.96892.

#### 3.1.5. 6-Bromo-7-(furan-2-yl)pyrido[2,3-*d*]pyrimidine-2,4(1*H*,3*H*)-dione (**8**)

To a solution of **7** (100 mg, 0.35 mmol) in 2 mL dimethyl sulfoxide (DMSO), sodium hydride (35.4 mg, 0.88 mmol, 60% in oil) was added and the mixture was stirred for 30 min at room temperature. Afterwards, *N,N*-carbonyldiimidazole (CDI, 60 mg, 0.39 mmol) dissolved in 1 mL *N,N*-dimethylformamide (DMF) was added and the reaction mixture heated at 90 °C for two hours. After cooling to room temperature, the solution was treated with 100 mL aqueous 1M HCl and extracted with ethyl acetate (4 × 25 mL). The combined organic phases were dried over sodium sulfate, filtered, and the solvent was removed under reduced pressure. The obtained residue was purified by column chromatography (SiO_2_, CH_2_Cl_2_/CH_3_OH, 39/1, *v*/*v*) to obtain **8** with a yield of 50% (55 mg, 0.18 mmol).

^1^H NMR (DMSO-*d_6_*, 400 MHz) *δ*: 6.78 (dd, *J* = 3.6, 1.8 Hz, 1 H), 7.58 (d, *J* = 3.6 Hz, 1 H), 8.04 (d, *J* = 1.4 Hz, 1 H), 8.40 (s, 1 H), 11.55 (s, 1H), 11.87 (s, 1 H) ppm. ^13^C NMR (DMSO-*d_6_*, 100 MHz) *δ*: 108.4, 110.2, 112.9, 117.0, 142.0, 146.5, 149.5, 149.7, 150.2, 151.2, 161.5 ppm. HRMS (ESI-) *m/z* calcd. for C_11_H_5_O_3_N_3_^79^Br^-^ [M-H]^-^ 305.95198, found 305.95750.

#### 3.1.6. 6-(2-Fluoropyridin-4-yl)-7-(furan-2-yl)pyrido[2,3-*d*]pyrimidine-2,4(1*H*,3*H*)-dione (**9**)

Compound **8** (50 mg, 0.16 mmol) was dissolved in 5 mL 1,4-dioxane together with 0.8 mL (1.60 mmol) of a 2 M Cs_2_CO_3_ solution and 2-(fluoropyridin-4-yl)boronic acid (34 mg, 0.24 mmol). The mixture was evacuated three times under reduced pressure and refilled with argon to remove air. Subsequently, [Pd(dppf)Cl_2_] (12 mg, 0.016 mmol) was added under argon and the resulting suspension was stirred at 90 °C for two hours. After cooling to room temperature, the solution was treated with 100 mL aqueous 1 M HCl and extracted with ethyl acetate (4 × 25 mL). The combined organic phases were dried over sodium sulfate, filtered, and the solvent was removed under reduced pressure. The obtained residue was pre-purified by column chromatography (SiO_2_, CH_2_Cl_2_/CH_3_OH/ethyl acetate, 99/1/1, *v*/*v/v*) to obtain 31 mg (0.06 mmol, 59%) of crude **9**, which was then purified by semi-preparative HPLC (Reprosil-Pur Basic C18-HD, 250 × 20 mm, 35% ACN/aq. 20 mM NH_4_OAc, flow 7.5 mL/min, t_R_ ~ 17–19 min).

^1^H NMR (DMSO-*d_6_*, 400 MHz) *δ*: 6.60 (d, *J* = 3.6 Hz, 1H), 6.75 (d, *J* = 3.5 Hz, 1H), 7.24 (s, 1H), 7.29 (d, *J* = 5.1 Hz, 1H), 7.74 (s, 1H), 8.13 (s, 1H), 8.27 (d, *J* = 5.2 Hz, 1H), 11.71 (bs, 2H) ppm. ^13^C NMR (DMSO-d_6_, 100 MHz) *δ*: 108.6, 110.2 (d, ^2^*J_C,F_* = 38.5 Hz), 112.9, 115.4, 123.0 (d, ^4^*J_C,F_* = 3.9 Hz), 125.7 (d, ^4^*J_C,F_* = 3.5 Hz), 139.3, 146.1, 147.8 (d, ^3^*J_C,F_* = 15.7 Hz), 149.6, 150.9, 151.4, 152.4, 152.8 (d, ^3^*J_C,F_* = 8.4 Hz), 162.1, 163.7 (d, ^1^*J_C,F_* = 235.3 Hz) ppm. ^19^F NMR (DMSO-*d_6_*, 376 MHz) *δ*: -68.6 ppm. HRMS (ESI-) *m/z* calcd. for C_16_H_8_O_3_N_4_F^-^ [M-H]^-^ 323.05859, found 323.05896.

#### 3.1.7. 7-(Furan-2-yl)-6-(2-nitropyridin-4-yl)pyrido[2,3-*d*]pyrimidine-2,4(1*H*,3*H*)-dione (**10**)

4-Bromo-2-nitropyridine (200 mg, 1.0 mmol), 4,4,4’,4’5,5,5’,5’-octamethyl-2,2’-bis(1,3,2-dioxaborolane) (250 mg, 1.0 mmol), KOAc (560 mg, 5.0 mmol), and [Pd(dppf)Cl_2_] (37 mg, 0.05 mmol) were suspended in 2 mL 1,4-dioxane under an inert atmosphere and the reaction mixture was heated to 90 °C. After an hour, the reaction mixture was cooled to room temperature and compound **8** (307 mg, 1.0 mmol), [Pd(dppf)Cl_2_] (37 mg, 0.05 mmol), and Cs_2_CO_3_ (3.25 g, 10.0 mmol) were added under inert atmosphere and heated for an hour at 90 °C. After cooling to room temperature, the suspension was filtered over celite, the solvent was removed under reducer pressure, and the residue was purified by column chromatography (SiO_2_, CH_2_Cl_2_/CH_3_OH, 99.5/0.5 to 98/2, *v*/*v*) to give **10** with a yield of 9% (31 mg, 0.088 mmol).

^1^H NMR (DMSO-*d*_6,_ 400 MHz) *δ*: 11.95 (s, 1H), 11.58 (s, 1H), 8.72 (d, *J* = 4.9 Hz, 1H), 8.31 (d, *J* = 1.4 Hz, 1H), 8.25 (s, 1H), 7.88 (dd, *J* = 4.9, 1.5 Hz, 1H), 7.71 (d, *J* = 1.6 Hz, 1H), 6.88 (d, *J* = 3.5 Hz, 1H), 6.62 (dd, *J* = 3.5, 1.7 Hz, 1H) ppm. ^13^C NMR (DMSO-*d*_6,_ 100 MHz) *δ*: 162.1, 157.2, 152.6, 151.4, 151.3, 150.9, 149.6, 149.2, 146.3, 139.9, 130.6, 124.9, 118.9, 115.6, 113.0, 108.7 ppm. HRMS (ESI+): *m/z* calcd. for C_16_H_9_N_5_NaO_5_ [M+Na]^+^ 374.0496, found 374.0506.

### 3.2. In Vitro Radioligand Binding Experiments

Membrane preparations of recombinant CHO or HEK (human embryonic kidney) cells expressing the respective human adenosine receptor subtype were obtained according to Borrman et al. [26] or purchased from Perkin Elmer, Germany. Radioligand binding assays using human A_1_, A_2A_, A_2B_ and A_3_ receptors were performed according to Alnouri et al. [27].

The following radioligands were employed:

[^3^H]2-Chloro-N^6^cyclopentyladenosine ([^3^H]CCPA, molar activity: 58 Ci/mmol, 1 nM [28]) for the A_1_ receptor,

[^3^H]3-(3-hydroxypropyl)-7-methyl-8-(*m*-methoxystyryl)-1-propargylxanthine, ([^3^H]MSX-2, molar activity: 84 Ci/mmol, 1 nM [29]) for the A_2A_ receptor,

[^3^H]8-(4-[4-(4-chlorophenyl)piperazine-1-sulfonyl]phenyl)-1-propyl-2,3,6,7-tetrahydro-1*H*-purine-2,6-dione ([^3^H]PSB-603, molar activity: 73Ci/mmol, 0.3 nM [26]) for the A_2B_ receptor, and

[^3^H](*R*)-8-ethyl-4-methyl-2-(phenyl)1,4,7,8-tetrahydro-5*H*-imidazo[2,1-*i*]purin-5-one ([^3^H]PSB-11, molar activity: 53 Ci/mmol, 1 nM [30]) for the A_3_ receptor. Three separate experiments were performed to determine the *K*_i_ values. All data were analyzed with GraphPad Prism, Version 4.1 (GraphPad Inc., La Jolla, CA,USA).

### 3.3. Radiochemistry

#### 3.3.1. General

No-carrier-added [^18^F]fluoride was produced via the [^18^O(p,n)^18^F] nuclear reaction by irradiation of an [^18^O]H_2_O target (Hyox 18 enriched water, Rotem Industries Ltd, Arava, Israel) on a Cyclone 18/9 (IBA RadioPharma Solutions, Lourain-La-Neuve, Belgium) with a fixed energy proton beam using a Nirta [^18^F]fluoride XL target.

Analytical chromatographic separations were performed on a JASCO LC-2000 system, incorporating a PU-2080*Plus* pump, AS-2055*Plus* auto injector (100 μL sample loop), and a UV-2070*Plus* detector (JASCO Deutschland GmbH, Pfungstadt, Germany) coupled with a gamma radioactivity HPLC detector (Gabi Star, raytest Isotopenmessgeräte GmbH, Straubenhardt, Germany). Data analysis was performed with Galaxie chromatography software (Agilent Technologies Deutschland GmbH, Waldbronn, Germany) using the chromatograms obtained at 254 nm. A Reprosil-Pur C18-AQ column (250 × 4.6 mm; 5 µm; Dr. Maisch HPLC GmbH; Ammerbuch-Entingen, Germany) with ACN/20 mM NH_4_OAc aq. (pH 6.8) as eluent mixture and a flow of 1.0 mL/min was used (gradient: eluent A 10% ACN/20 mM aq. NH_4_OAc; eluent B 90% ACN/20 mM aq. NH_4_OAc; 0–5 min 100% A, 5–25 min up to 50% B, 25–26 min up to 100% B, 26–30 min 100% B, 30–31 min up to 100% A and 31–40 min 100% A).

The ammonium acetate and sodium dodecyl sulfate (SDS) concentrations stated as aq. 20 mM NH_4_OAc and aq. 100 mM, respectively, correspond to the concentrations in the aqueous component of an eluent mixture.

#### 3.3.2. Radiosynthesis

Remote-controlled radiosynthesis of **[^18^F]9** was performed using a TRACERlab FX2 N synthesizer (GE Healthcare, Waukesha, WI, USA) equipped with a Laboport vacuum pump N810.3FT.18 (KNF Neuburger GmbH, Freiburg, Germany), a BlueShadow UV detector 10D (KNAUER GmbH, Berlin, Germany), and the TRACERlab FX Software (GE Healthcare, Waukesha, WI, USA).

**Figure 4:**
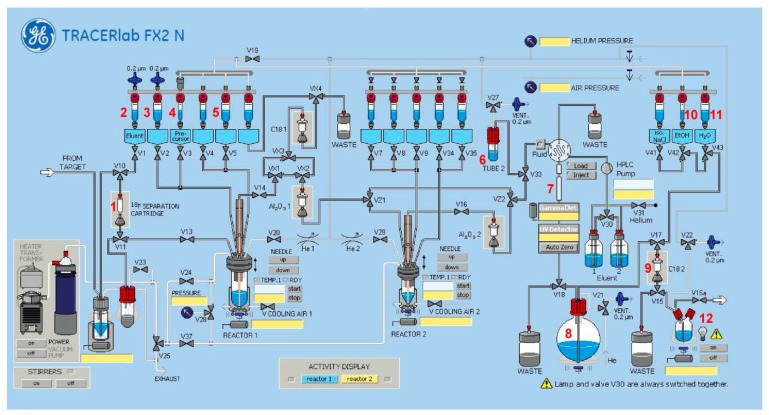
Scheme of the synthesis module TRACERlab FX2 N for the radiosynthesis of **[^18^F]9**. (1) Sep-Pak Accell Plus QMA Carbonate Plus light, (2) K_2_CO_3_ (1.8 mg in 400 µL water), (3) K_2.2.2._ (11 mg in 1.5 mL ACN), (4) precursor (0.5 mg of **10** in 800 µL DMSO), (5) 1.5 mL water and 2.0 mL MeOH/water, (6) injection vial, (7) Reprosil-Pur 120 AQ (60% MeOH/aq. 20 mM NH_4_OAc, flow 8.0 mL/min), (8) 40 mL water, (9) Sep-Pak^®^ C18 light, (10) 2 mL water, (11) 1.3 mL EtOH, (12) product vial.

[^18^F]Fluoride (2–7 GBq) was trapped on a Sep-Pak Accell Plus QMA Carbonate Plus light cartridge (Figure 4, entry **1**, Waters GmbH, Eschborn, Germany) and eluted into the reactor with 400 µL of an aqueous solution of potassium carbonate (K_2_CO_3_, 1.8 mg, 13 µmol, entry **2**). After addition of Kryptofix 2.2.2. in 1.5 mL ACN (11 mg, 29 µmol, entry **3**), the mixture was azeotropically dried for approximately 10 min. Thereafter, 0.5 – 0.6 mg of the nitro precursor (**10**) dissolved in 800 µL DMSO (entry **4**) was added, and the reaction mixture was stirred at 120 °C for 15 min. After cooling, the reaction mixture was diluted with 1.5 mL H_2_O and 2.0 mL MeOH/H_2_O 1/1 (*v*/*v*, entry **5**) and transferred into the injection vial (entry **6**). Semi-preparative HPLC was performed using a Reprosil-Pur 120 AQ column (250 × 20 mm; 10 µm; Dr. Maisch HPLC GmbH; Ammerbuch-Entingen, Germany) with a solvent composition of 60% MeOH/aq. 20 mM NH_4_OAc at a flow rate of 8.0 mL/min (entry **7**). **[^18^F]9** was collected in the dilution vessel (entry **8)** previously loaded with 40 mL H_2_O. Final purification was performed by passing the solution through a Sep-Pak^®^ C18 light cartridge (entry **9**), followed by washing with 2 mL water (entry **10**) and elution of **[^18^F]9** with 1.3 mL EtOH (entry **11**) into the product vial (entry **12**). The ethanolic solution was transferred out of the hot cell and the solvent was reduced under a gentle argon stream at 70 °C to a final volume of 10–50 µL. Afterwards, the radiotracer was diluted in isotonic saline to obtain a final product containing 10% of EtOH (*v*/*v*).

The molar activity was determined on the basis of a calibration curve created under isocratic HPLC conditions (22% ACN/aq. 20 mM NH_4_OAc; Reprosil-Pur 120 AQ 250 × 4.6 mm, flow 1.0 mL/min), using chromatograms obtained at 254 nM as an appropriate maximum of UV absorbance.

The partition coefficient of **[^18^F]9** was experimentally determined for the *n*-octanol/PBS system by the shake-flask method as described previously [14].

### 3.4. In Vivo Studies in Mice

For metabolism studies (*n* = 2), **[^18^F]9** (30–40 MBq) was administered as a bolus in awake female CD-1 mice (10–12 weeks, 25–32 g) via the tail vein. At 30 min p.i., the animals were slightly anesthetized with isoflurane, and blood was sampled from retro-orbital plexus. Blood plasma was obtained as a supernatant after centrifugation of the whole blood samples (14,000 rpm, 1 min; Centrifuge 5418, Eppendorf Vertrieb Deutschland GmbH, Wesseling-Berzdorf, Germany). After cervical luxation of the animals, urine samples were obtained. All samples were weighed and the respective activity measured in a dose calibrator (ISOMED 2010; MED Nuklear-Medizintechnik Dresden GmbH, Dresden, Germany).

MLC: For preparation of the MLC samples, mouse plasma (20–50 µL) was dissolved in 200 µL of 200 mM aq. sodium dodecyl sulfate (SDS) and injected into the MLC system. The MLC system was built up as described previously [14]. Separations were performed using a Reprosil-Pur C18-AQ column (250 × 4.6 mm + 10 mm pre-column, particle size: 10 µm) and an eluent mixture of EtOH/100 mM aq. SDS/25 mM (NH_4_)_2_HPO_4_ in gradient mode (0–10 min at 100% 100 mM aq. SDS, 10–15 min up to 10% EtOH, 15–20 min at 10% EtOH, 20–21 min up to 100% 100 mM aq. SDS; 21–30 min at 100% 100 mM aq. SDS) at a flow rate of 1.0 mL/min. The same HPLC apparatus was used as described in the radiochemistry part in generals.

RP-HPLC: Protein precipitation was performed by addition of an ice-cold mixture of ACN/water (9/1; *v*/*v*) in a ratio of 4:1 (*v*/*v*) of organic solvent to plasma. The sample workup was performed according to a procedure previously described in detail [14]. The urine samples were diluted with water and directly injected. To analyze the samples, the same HPLC apparatus and method was used as described in the radiochemistry section.

The biodistribution of the radiotracer was assessed in female CD-1 mice (30.6 ± 0.4 g bodyweight) using a dedicated small-animal PET-MRI scanner (nanoScan, Mediso, Hungary) over 60 min followed by a subsequent T1-weighted MR. The anaesthetized (2% isoflurane, carrier gas mixture of 40% air and 60% O_2_) female CD-1 mice were kept on a heated animal bed to maintain body temperature during imaging. Vehicle (10% DMSO in isotonic 0.9% NaCl solution) or compound **2** at 1 mg/kg bodyweight were co-injected with the tracer (**[^18^F]9**: injected dose of 6.9 ± 0.1 MBq, equivalent to 10.4 ± 0.9 fmol/g bodyweight) intravenously 20 seconds after starting the dynamic PET acquisition, performed in normal mode and with a coincidence mode 1–5. For subsequent dynamic reconstructions (Nucline v2.01, Mediso, Hungary) by Ordered Subset Expectation Maximization (OSEM3D) with an attenuation correction with four iterations, six subsets, and a voxel size of 0.4 mm^3^, list mode data were sorted into sinograms (12 × 10 s, 6 × 30 s, 5 × 60 s, and 10 × 300 s). PMOD software (v4.005, PMOD Technologies LLC, Zurich, Switzerland) was used for analysis of reconstructed studies and the results are expressed as standardized uptake value (SUV) given in mean ± SD.

Animals for in vivo studies were obtained from the Medizinisch-Experimentelles Zentrum, Universität Leipzig. All procedures that included animals were approved by the respective State Animal Care and Use Committee and conducted in accordance with the German Law for the Protection of Animals (TVV 18/18 since 20 June 2018, DD24.1-5131/446/19, Landesdirektion Sachsen).

## Figures and Tables

**Table 1:** *K*_i_ values of **1**, lead compound **2**, and the new fluorinated derivative **9** at the four human adenosine receptor subtypes along with the corresponding selectivity ratios.

Compound	*K*_i_ in nM ^a^				Selectivity Ratio *K*_i_(A_x_)*/K*_i_(A_2B_)			
	A_2B_	A_2A_	A_1_	A_3_		A_2A_/A_2B_	A_1_/A_2B_	A_3_/A_2B_
**1**	4.24 ± 0.04 ^b^	55.0 ± 6.1 ^b^	19.0 ± 5.2 ^b^	796 ± 26 ^b^		13	4.5	188
**2**	2.51 ± 1.1	98.8 ± 28.3	> 1000	> 1000		39	> 400	> 400
	1 ± 0 ^c^	181 ± 25 ^c^	1727 ± 617 ^c^	6267 ± 2322 ^c^				
**9**	2.51 ± 0.58	107 ± 15	149 ± 26	286 ± 10		43	59	114

^a^ Data are means ± SEM of three independent assays, each performed in duplicate. Competitive binding assays with [^3^H]PSB-603 (A_2B_), [^3^H]MSX-2 (A_2A_), [^3^H]CCPA (A_1_), and [^3^H]PSB-11 (A_3_) as radioligands and membranes obtained from CHO or HEK cells stably expressing the corresponding human adenosine receptor. ^b,c^ Data are taken from References [14,16], resp.

## Supplementary Materials

Supplementary materials can be found at ’https://www.mdpi.com/1422-0067/21/9/3197/s1. Figure S1: Semi-preparative radio- and UV-HPLC chromatograms of **[^18^F]9**. Figure S2: Analytical radio- and UV-HPLC chromatograms of the final product of **[^18^F]9** spiked with the non-radioactive reference **9.** Figure S3. Maximum Intensity Projections (MIP) of mice treated with vehicle and compound **2** coinjected with **[^18^F]9**.
