# Supplementary material for: Development of a Radiofluorinated Adenosine A2B Receptor Antagonist as Potential Ligand for PET Imaging
Source: Int J Mol Sci. 2020 Apr 30;21(9):3197. doi: 10.3390/ijms21093197 (PMC7246765; doi:10.3390/ijms21093197)
Supplement: Supplementary file 1 [file ijms-21-03197-s001.pdf]

International Journal of
Molecular Sciences

MDPI

Supplementary Material

1. Syntheses and analytical data of the intermediates 3–6

*1.1. 3-(Dimethylamino)-1-(furan-2-yl)prop-2-en-1-one (**3**)*

To 16 mL (120.8 mmol) *N*,*N*-dimethylformamide dimethyl acetal were added 10 g of acetyl furane (90.82 mmol) and the mixture was refluxed for 5.5 h. After cooling to room temperature the solvent was removed under reduced pressure. The residue was dissolved in diethyl ether and the desired product was precipitated by adding 150 mL *n*-hexane. The precipitate was filtered off and washed with 50 mL diethyl ether/*n*-hexane (1:1, *v*/*v*). After drying under reduced pressure, 13.5 g (81.9 mmol, 90%) of crude product **3** was obtained, which was used without further purification for the following reactions.

^{1}H NMR (DMSO-d$_6$, 300 MHz) δ: 7.79 (dd, *J* = 1.8, 0.8 Hz, 1 H), 7.69 (d, *J* = 12.4 Hz, 1 H), 7.10 (dd, *J* = 3.4, 0.8 Hz, 1H), 6.60 (dd, *J* = 3.4 Hz, 1 H), 5.65 (d, *J* = 12.4 Hz, 1 H), 3.13 (s, 3 H), 2.88 (s, 3 H) ppm. ^{13}C NMR (DMSO-d$_6$, 100 MHz) δ: 175.9, 155.0, 153.7, 145.3, 113.5, 112.3, 91.1, 44.9, 37.5 ppm. HRMS (ESI+) *m*/*z* calcd. for $C_9H_{12}O_2N^+$ [M+H]$^+$ 166.08626, $C_9H_{11}O_2NNa^+$ [M+Na]$^+$ 188.06820, found 166.08521, 188.06683.

*1.2. 2-Cyanoethanimidic acid methyl ester hydrochloride (**4**)*

A mixture of malononitrile (24.8 g, 0.37 mol) and 15.2 mL methanol in 500 mL diethyl ether was cooled to 0 °C and a slight stream of dry HCl gas was added over 2.5 h. Afterwards, 500 mL CH_2Cl_2 was added, the formed precipitate was filtered off, washed with 250 mL CH_2Cl_2/diethyl ether (1:1) and dried under vacuum. Compound **4** was obtained as an off-white powder with a yield of 54 % (27.0 g, 0.20 mol). Melting point: 103–106 °C.

*1.3. 2-Amino-6-(furan-2-yl)nicotinonitrile (**5**)*

Compound **5** was synthesized with slight modifications according to the reported procedure of Troschütz et al. [1].

Compound **4** (1.6 g, 12.1 mmol) and ammoniumacetate (3.3 g, 42.4 mmol) were dissolved in 50 mL MeOH and refluxed for 15 min. Then, the furan **3** (2.0 g, 12.1 mmol) was added and the mixture refluxed for further 3.5 hours. After removing the solvent under reduced pressure, the residue was quenched with 100 mL water, the precipitate filtered off, washed with 50 mL water and dried in a vacuum desiccator (over calcium chloride) to obtain 1.6 g (8.5 mmol, 70 %) of the crude product **5**.

^{1}H NMR (DMSO-d$_6$, 300 MHz) δ: 6.64 (dd, *J* = 3.4, 1.7 Hz, 1 H), 6.91 (bs, 2 H), 6.98 (d, *J* = 8.1 Hz, 1 H), 7.10 (dd, *J* = 3.5, 0.8 Hz, 1 H), 7.84 (dd, *J* = 1.8, 0.8 Hz, 1 H), 7.88 (d, *J* = 8.0 Hz, 1H) ppm. ^{13}C NMR (DMSO-d$_6$, 75 MHz) δ: 88.0, 107.2, 111.8, 113.0, 117.7, 143.5, 145.7, 151.2, 152.7, 160.1 ppm. HRMS (ESI+) *m*/*z* calcd. for $C_{10}H_8ON_3^+$ [M+H]$^+$ 186.06619, found 186.06757.

*1.4 2-Amino-5-bromo-6-(furan-2-yl)nicotinonitrile (**6**)*

Compound **6** was synthesized with slight modifications according to the reported procedure of Harada et al. [2].

Compound **5** (1.4 g, 7.56 mmol) and 2.5 g (14.04 mmol) of *N*-bromosuccinimide were dissolved in 30 mL DMF and stirred for 12 hours at room temperature. The solution was then poured into 200 mL ethyl acetate and extracted with saturated aqueous sodium carbonate solution (2 x 500 mL) and with 250 mL brine. The combined organic layers were dried over sodium sulfate, filtered and the solvent was evaporated under reduced pressure. The crude material was purified by column chromatography (silica gel, ethyl acetate/*n*-hexane 1:2 *v*/*v*) to give 1.5 g (5.80 mmol, 77%) of compound **6**.

R_f: 0.45 (ethyl acetate/*n*-hexane 1 : 2 *v*/*v*, silica gel). ^{1}H NMR (DMSO-d6, 400 MHz) δ: 6.72 (dd, *J* = 3.5, 1.7 Hz, 1 H), 7.17 (s, 2 H), 7.44 (dd, *J* = 3.5, 0.8 Hz, 1 H), 7.95 (dd, *J* = 1.7, 0.8 Hz, 1 H), 8.26 (s, 1 H) ppm. ^{13}C NMR (DMSO-d6, 100 MHz) δ: 90.2, 100.1, 112.6, 116.0, 116.1, 145.8, 147.6, 148.6, 150.5, 158.2 ppm. HRMS (ESI+) *m*/*z* calcd. for $C_{10}H_7ON_3{}^{79}Br^+$ $[M+H]^+$ 263.97670, found 263.97864.

2. ^{1}H and ^{13}C NMR Spectra of Compounds 2, 9 and 10

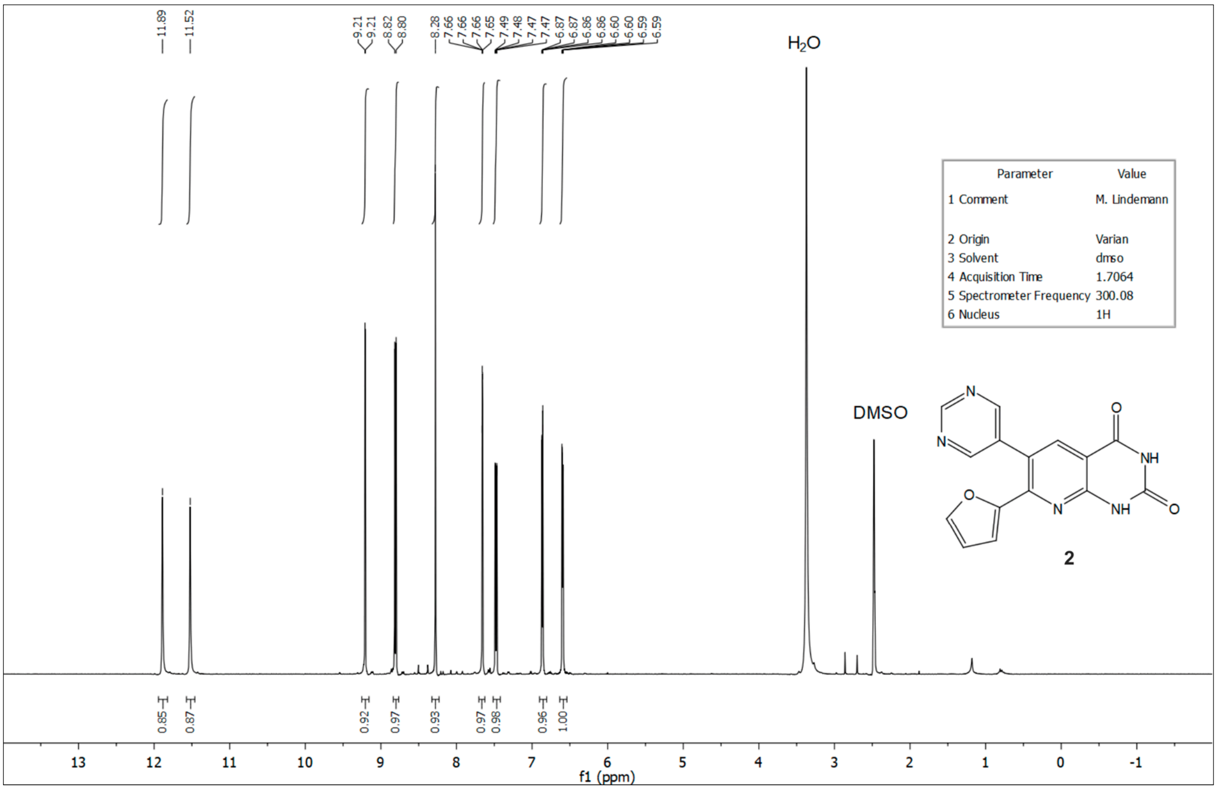

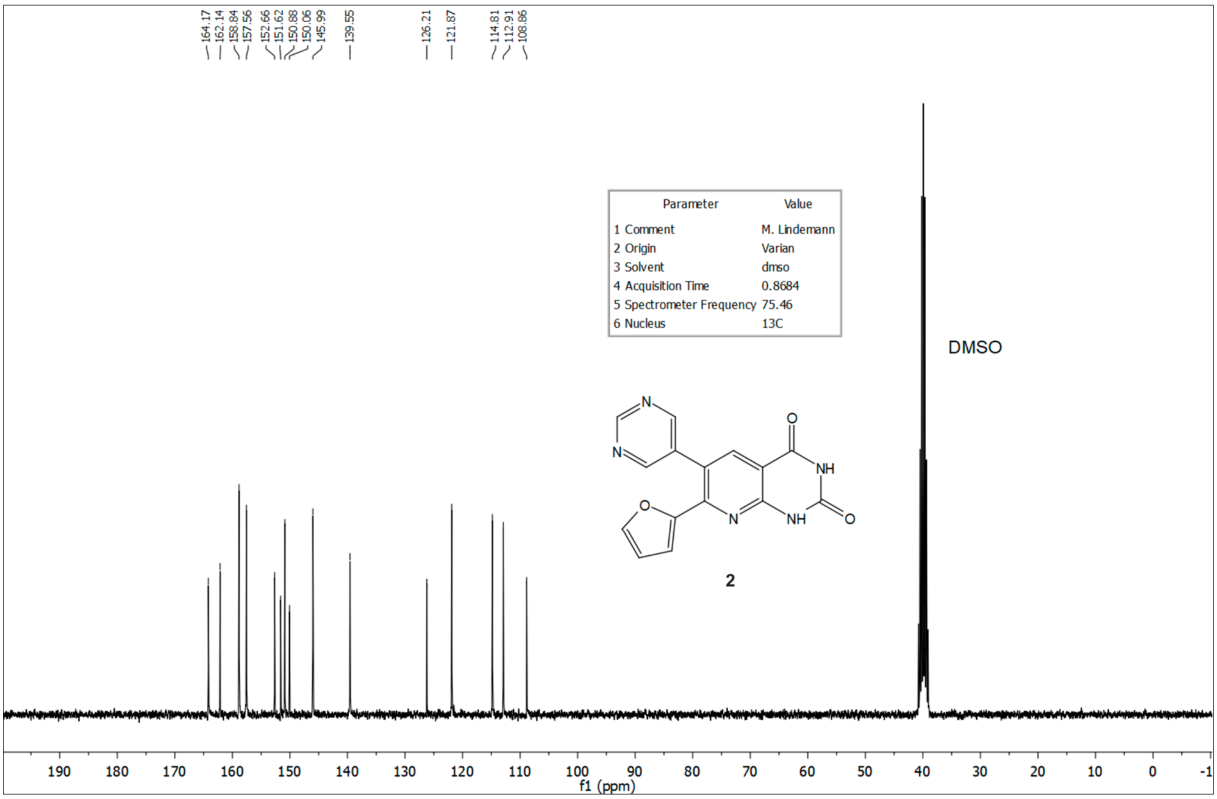

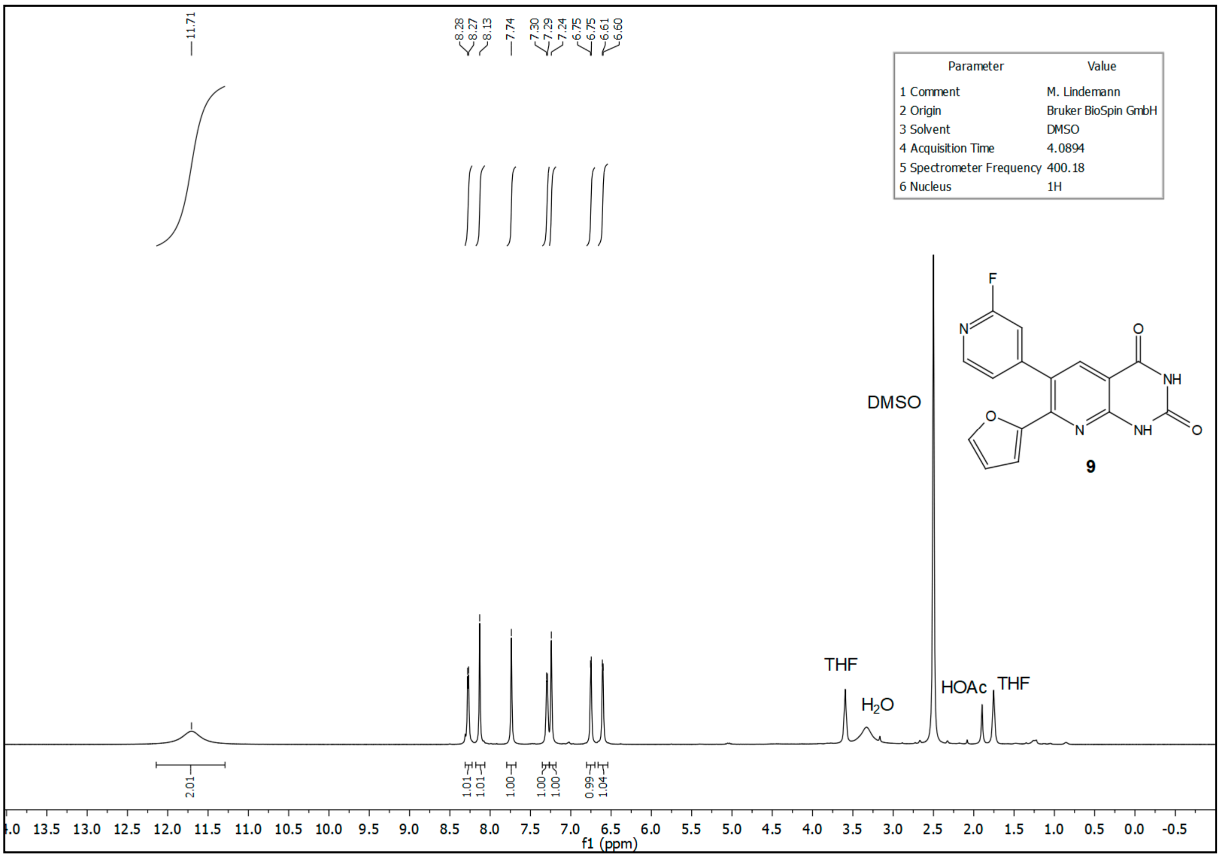
11.71
8.28
8.27
8.13
7.74
7.30
7.29
7.24
6.75
6.75
6.61
6.60
Parameter Value
1 Comment M. Lindemann
2 Origin Bruker BioSpin GmbH
3 Solvent DMSO
4 Acquisition Time 4.0894
5 Spectrometer Frequency 400.18
6 Nucleus 1H
F
N
O
NH
NH
N
O
O
9
DMSO
THF
H2O
HOAc
THF
2.01
1.01
1.01
1.00
1.00
1.00
0.99
1.04
13.5 13.0 12.5 12.0 11.5 11.0 10.5 10.0 9.5 9.0 8.5 8.0 7.5 7.0 6.5 6.0 5.5 5.0 4.5 4.0 3.5 3.0 2.5 2.0 1.5 1.0 0.5 0.0 -0.5
f1 (ppm)

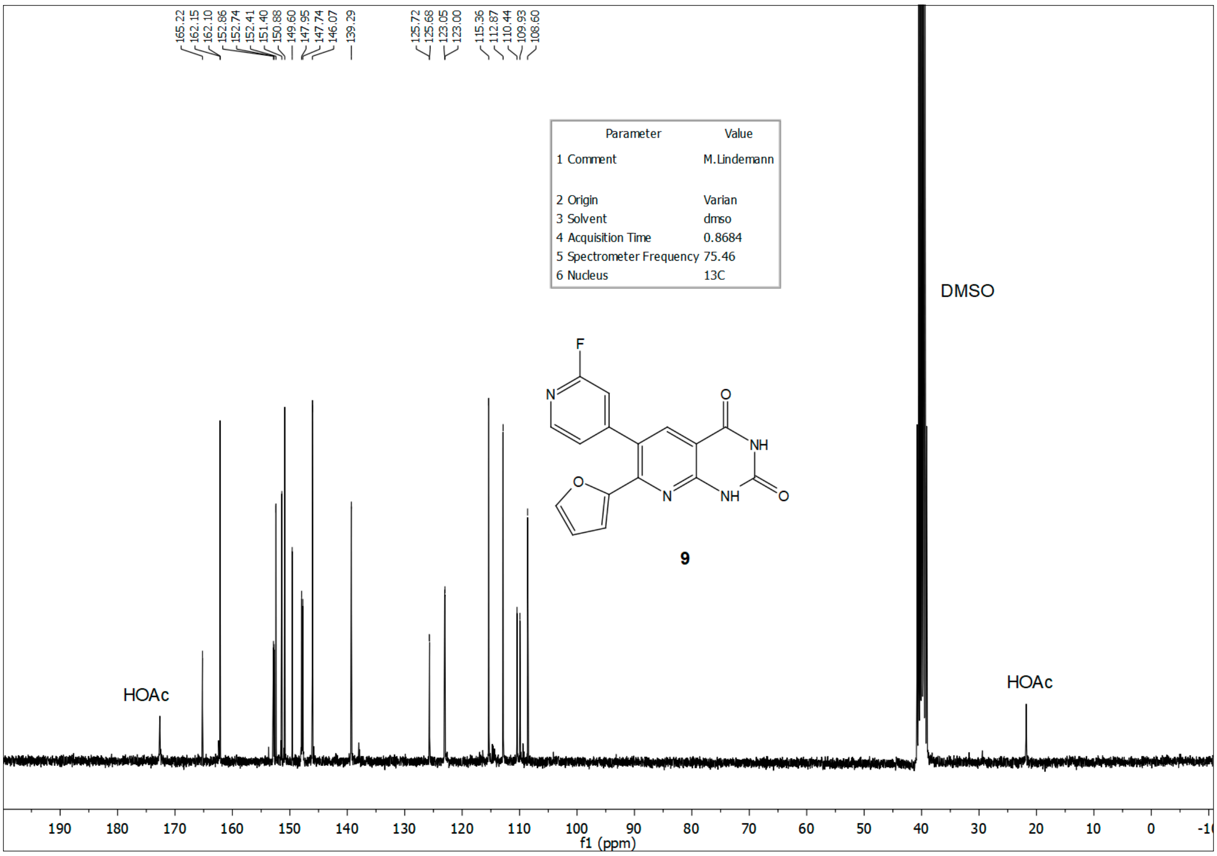
165.22
162.15
162.10
152.86
152.74
152.41
151.40
150.88
149.60
147.95
147.74
146.07
139.29
125.72
125.68
123.05
123.00
115.36
112.87
110.44
109.93
108.60
Parameter Value
1 Comment M.Lindemann
2 Origin Varian
3 Solvent dmso
4 Acquisition Time 0.8684
5 Spectrometer Frequency 75.46
6 Nucleus 13C
DMSO
F
N
O
NH
NH
N
O
O
9
HOAc
HOAc
190 180 170 160 150 140 130 120 110 100 90 80 70 60 50 40 30 20 10 0
f1 (ppm)

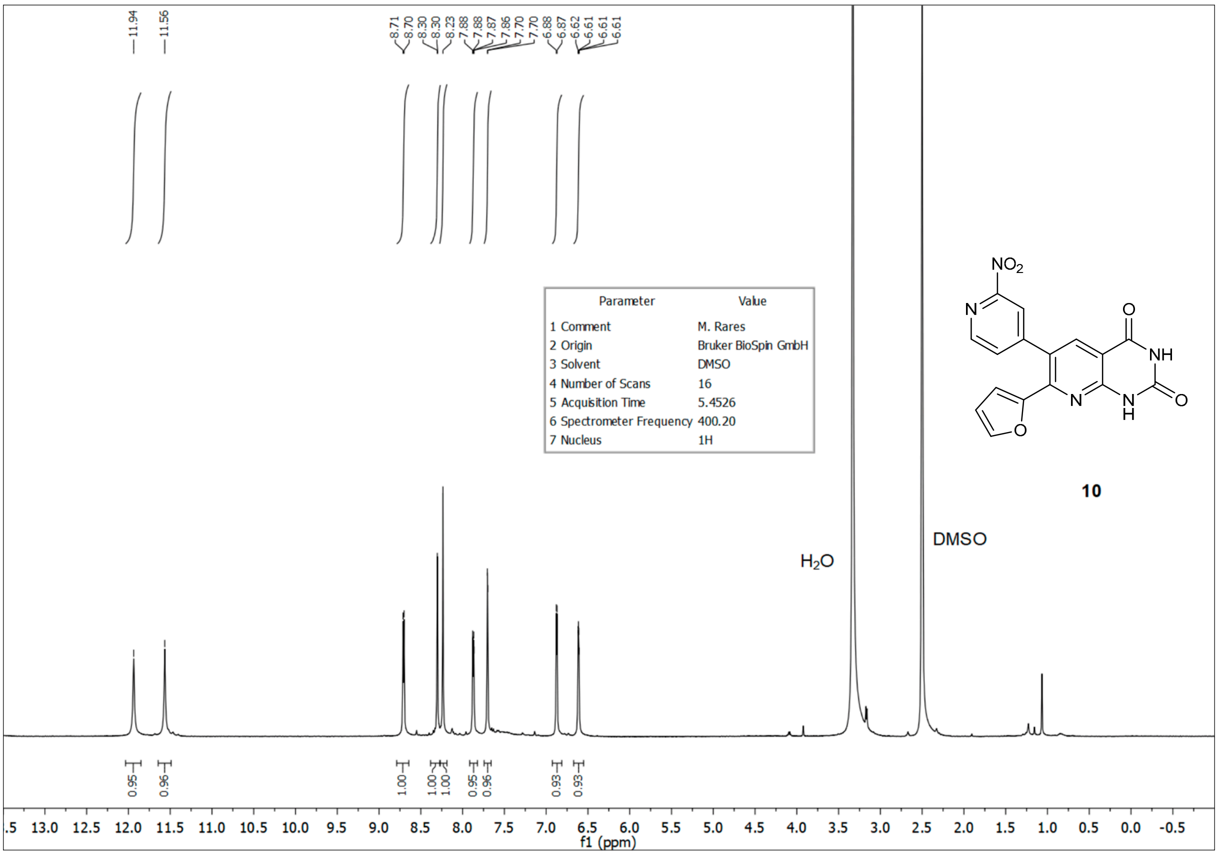
11.94
11.56
8.71
8.70
8.30
8.30
8.23
7.88
7.88
7.87
7.86
7.70
7.70
6.88
6.87
6.62
6.61
6.61
6.61
Parameter Value
1 Comment M. Rares
2 Origin Bruker BioSpin GmbH
3 Solvent DMSO
4 Number of Scans 16
5 Acquisition Time 5.4526
6 Spectrometer Frequency 400.20
7 Nucleus 1H
NO2
N
O
NH
N
N
H
O
O
10
H2O
DMSO
0.95
0.96
1.00
1.00
1.00
0.95
0.96
0.93
0.93
f1 (ppm)

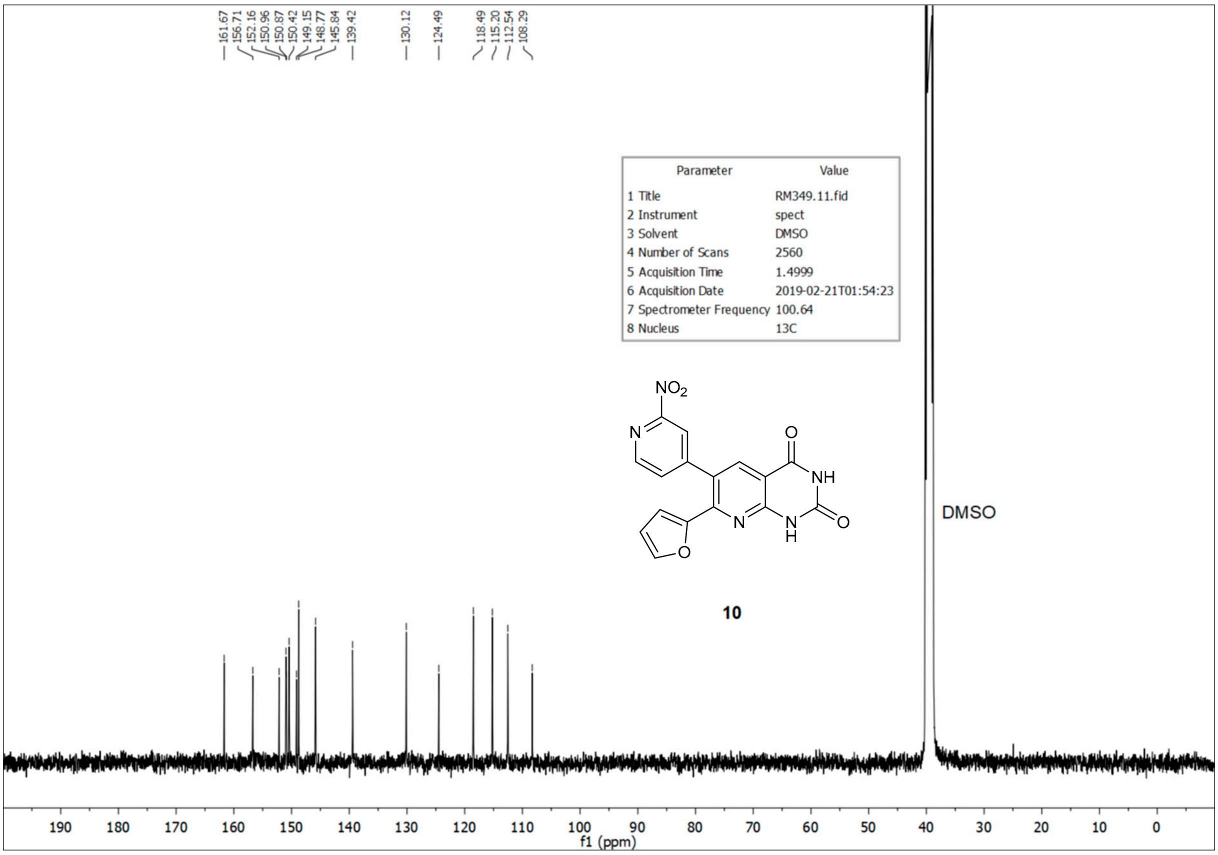
161.67
156.71
152.16
150.96
150.87
150.42
149.15
148.77
145.84
139.42
130.12
124.49
118.49
115.20
112.54
108.29
Parameter Value
1 Title RM349.11.fid
2 Instrument spect
3 Solvent DMSO
4 Number of Scans 2560
5 Acquisition Time 1.4999
6 Acquisition Date 2019-02-21T01:54:23
7 Spectrometer Frequency 100.64
8 Nucleus 13C
NO2
N
O
NH
N
N
H
O
O
10
DMSO
f1 (ppm)

3. Representative Radio- and UV-HPLC Chromatograms of the Radiosynthesis of [18F]9

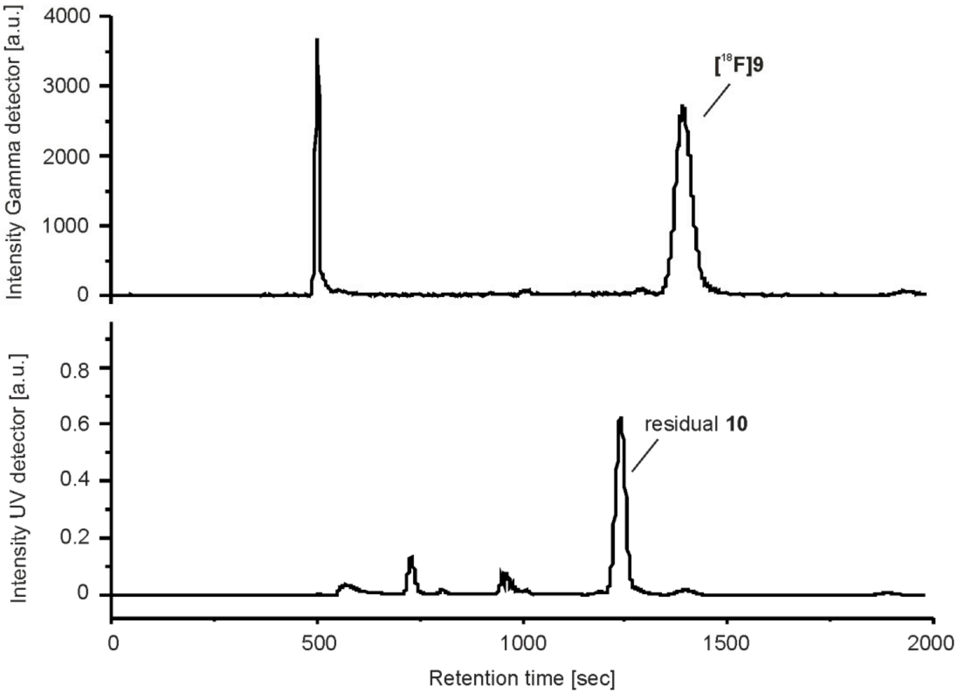

Figure S1. Semi-preparative radio- and UV-HPLC chromatograms of **[18F]9** (conditions: Reprosil-Pur C18 AQ, 250 x 20 mm, 60% MeOH/aqu. 20 mM NH_4OAc, 8.0 mL/min).

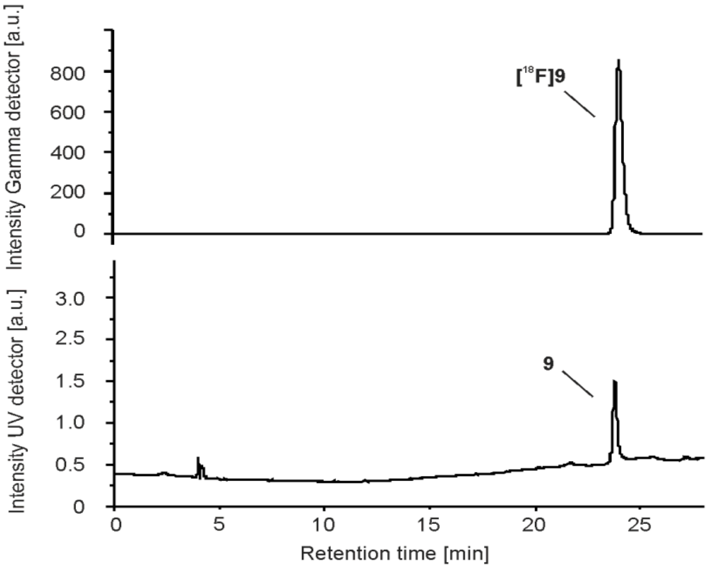

Figure S2. Analytical radio- and UV-HPLC chromatograms of the final product of **[18F]9** spiked with the non-radioactive reference **9** (conditions: Reprosil-Pur C18-AQ, 250 x 4.6 mm, gradient with an eluent mixture of ACN/aqu. 20 mM NH_4OAc, 1.0 mL/min).

4. PET/MR images of [^{18}F]9 in healthy mice

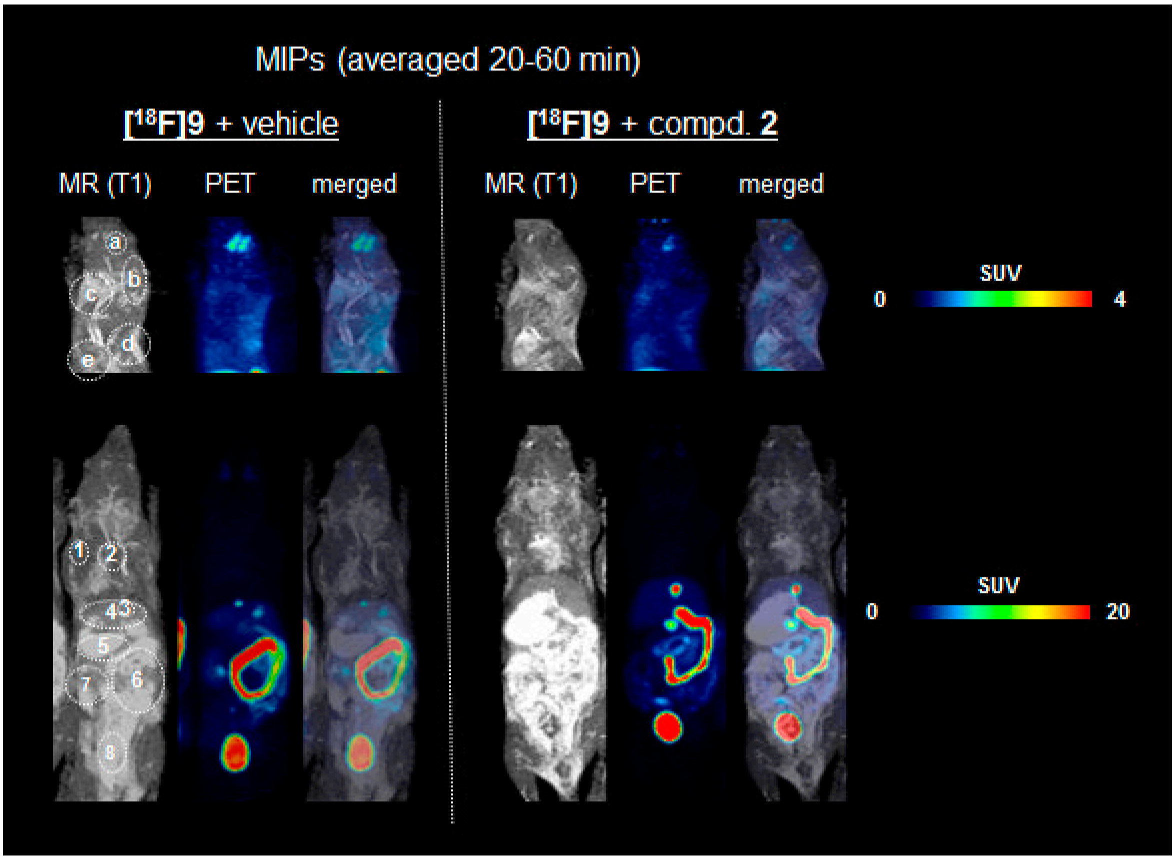

Figure S3. Maximum intensity projections (MIPs) of mice treated with vehicle (control group, left) and compound **2** (right) coinjected with **[^{18}F]9**. a) Harderian glands, b) brain, c) glandula mandibularis, d) brown adipose tissue, e) heart, 1) muscle, 2) thymus, 3) gall bladder, 4) liver, 5) stomach, 6) intestine, 7) left kidney, 8) bladder.

References

1. Troschütz, R.; Dennstedt, T. Synthesis of Substituted 2-Aminonicotinonitriles. *Arch. Pharm.* **1994**, *327*, 33-40.
2. Harada, H.; Asano, O.; Miyazawa, S.; Ueda, M.; Yasuda, M.; Yasuda, N. Aminopyridine compounds and use thereof as drugs. US 2004/0006082 A1 2004.
